# Perceptions and Experience of Patients Awaiting Orthopaedic Consultation for Hallux Valgus in Two Public Health Services in Australia

**DOI:** 10.1002/jfa2.70209

**Published:** 2026-09-12

**Authors:** Katja Veenendaal, Matthew Cotchett, Hylton B. Menz

**Affiliations:** ^1^ Community Care Program Canberra Health Services Canberra Australian Capital Territory Australia; ^2^ Discipline of Podiatry School of Allied Health, Human Services and Sport La Trobe University Melbourne Victoria Australia; ^3^ Northern Hospital Epping Melbourne Victoria Australia; ^4^ Faculty of Sports Science Chulalongkorn University Bangkok Thailand; ^5^ Center of Excellence in Exercise Physiology for Special Populations Chulalongkorn University Bangkok Thailand

**Keywords:** hallux valgus, podiatry, surgery, waiting lists

## Abstract

**Background:**

Hallux valgus (HV) is a common, progressive foot deformity associated with pain, functional limitation, and reduced quality of life. Patients are frequently referred for orthopaedic consultation contributing to prolonged waitlists. This study explored patient experiences, perceptions, and expectations of HV management while awaiting orthopaedic review.

**Methods:**

A convergent mixed‐methods design was used involving patients on orthopaedic waitlists at Canberra Health Services and Northern Health, Australia. Participants completed a survey assessing demographics, HV severity, health‐related quality of life, and psychological health, alongside semi‐structured interviews exploring lived experiences. Quantitative data were analysed descriptively, and qualitative data underwent applied thematic analysis.

**Results:**

Forty‐three participants completed the survey and eight participated in interviews. HV was associated with moderate pain, mobility limitations, and reduced health‐related quality of life. Most participants reported long symptom duration and worsening while waiting for specialist review. Qualitative findings revealed presence of chronic pain, footwear restriction, activity avoidance, and psychosocial burden. Participants described limited assessment and education in primary care, poor communication regarding waitlists, and uncertainty about management pathways. Although many believed surgery was necessary, fewer expected to proceed. Most expressed a preference for earlier assessment, education, and conservative management, and were receptive to podiatry‐led care.

**Conclusions:**

Patients with HV experience substantial unmet needs while awaiting orthopaedic consultation. Integrating podiatry‐led models may improve access to timely, patient‐centred care and reduce health system burden.

## 1 Background

Hallux valgus (HV) is a progressive deformity estimated to affect one in four people aged 18–65 years, and one in three people aged over 65 years [1]. HV is associated with foot pain, alterations in gait, and functional limitation [2, 3]. The condition has a negative impact on well‐being and health‐related quality of life, with greater HV severity associated with poorer physical function, general health, social functioning, and mental health outcomes [2]. In older adults, HV has been identified as a risk factor for falls, highlighting the broader functional and public health burden of the deformity [3].

People seeking treatment for symptoms of HV are frequently referred for orthopaedic consultation [4]. More than one‐third of HV patients have not accessed allied health care prior to orthopaedic referral [5]. This puts unnecessary strain on orthopaedic waitlists, and patients may miss valuable therapeutic windows for non‐surgical intervention while waiting to see a specialist [6]. While previous research has documented the clinical burden of HV and the effectiveness of some conservative interventions [6, 7, 8], there is limited evidence regarding patients' experiences of accessing and engaging with non‐surgical care. Existing qualitative research suggests that patients often perceive a lack of support, education, and treatment options within current care pathways [9]; however, little is known about how patients make decisions regarding surgery, the barriers they encounter when seeking conservative management, or how prolonged waits for orthopaedic consultation influence symptom burden, expectations, and healthcare needs. Consequently, there is insufficient patient‐centred evidence to inform the development of responsive and effective models of care for people with HV.

An advanced practice podiatrist‐led assessment service within a public orthopaedic unit at Northern Health (NH), Melbourne, Australia [10], found that HV was the most common diagnosis among foot and ankle referrals (22%), and many of these patients did not require an orthopaedic consultation (61%). In this context, “advanced practice” podiatrists are podiatrists with specialised knowledge and skills in the assessment, diagnosis, triage, and management of musculoskeletal foot and ankle conditions, enabling them to work in extended clinical roles within orthopaedic services. A subsequent qualitative study reported that patients with HV perceived a lack of support, education, and access to non‐surgical management options when managed by general practitioners [11]. The integration of advance practice podiatrists delivering non‐surgical interventions and patient education through triage and consultation models in Australian orthopaedic services has been associated with improved patient satisfaction, reduced surgical waiting list demand, enhanced surgical conversion rates, and cost‐effectiveness [9, 10, 12, 13, 14, 15, 16].

At Canberra Health Services (CHS), demand for orthopaedic assessment of foot conditions, including HV, has contributed to substantial waiting times. Median waiting times for orthopaedic consultation for foot conditions increased from 920 days in 2020 to 1634 days in 2024, with approximately 40% of referrals relating to HV. In response, CHS is exploring alternative models of care, including the introduction of an advanced practice podiatrist role within the orthopaedic pathway [12].

To inform the development of such service models, it is important to understand how patients experience HV and navigate existing care pathways. Greater insight into patients' views of non‐surgical management, their decision‐making regarding surgery, and their experiences while awaiting specialist review may help identify unmet needs and priorities for care. Although previous work from NH has highlighted some of these issues [9], findings may not be directly transferable to CHS because of differences in service delivery models, referral pathways, patient populations, and waiting list duration.

Accordingly, this study aimed to explore the experiences, needs, perceptions, and expectations of patients with HV currently on the orthopaedic waiting list at CHS and compare these findings with those from NH to better understand common and context‐specific aspects of the patient experience.

## 2 Methods

### 2.1 Ethics Approval

This study was approved by the ACT Health Human Research and Ethics Committee (2024.LRE.00317). Ethics approval was also obtained via cross institutional approval with Northern Health (SSA/116605/NH‐2025) and University of Canberra (UC) (14440). All participants provided informed consent prior to data collection.

### 2.2 Study Design and Reporting

This study used a convergent mixed methods design [17], where qualitative and quantitative data were collected and analysed in parallel, and then merged to compare and integrate findings. A mixed methods design was used to gain a more comprehensive, holistic, and nuanced understanding of the HV patient journey by bringing together both numeric trends through an online survey (quantitative) and detailed insights though participant interview (qualitative). The study is reported in accordance with the COnsolidated criteria for REporting Qualitative research (COREQ) framework [18] and the Good Reporting of a Mixed Methods Study (GRAMMs) Checklist [19].

### 2.3 Participant Recruitment

Patients on the CHS and NH orthopaedic non‐urgent waitlist during the period March to May 2025 and October to December 2025, respectively, who were over the age of 18 and had a referral for HV pain and/or deformity (unilateral and bilateral), were invited to participate in an online survey and/or interview. Patients were excluded from invitation only where the reason for referral was not clearly indicated as HV. No restrictions were placed on language as this information was not necessarily included on referrals and interpreters were available for participants with low English proficiency.

Patients received an SMS invitation which included a link to participant information and consent forms for the online survey. Patients were able to participate in the anonymous online survey once they had completed the electronic consent process. To maximise response rates, reminder SMS messages (three in total) were sent fortnightly to patients over a six‐week data collection period for each health service.

Participant recruitment for interviews occurred simultaneously via the SMS invitation. Patients were able to register their interest by calling or emailing the principal investigator, enabling participants to be involved in interview discussions regardless of survey completion. Participants who completed the online survey also received interview information at the end of the survey and were directed to a separate platform to register their details for interview participation if interested. CHS Allied Health Research Support Grant funding was used to provide interview participants a $100 gift voucher as a fair and proportionate reimbursement for their time and effort. This gift reflected standard compensation rates for interview participation without providing an undue inducement.

### 2.4 Sample Size

An a priori sample size calculation for the survey component estimated that 192 participants (112 CHS; 80 NH) were required, based on a total waitlist population of 254 patients (154 CHS; 100 NH), a 95% confidence level, a 5% margin of error, and an expected response rate of 40%. However, at recruitment, the number of eligible waitlisted individuals was lower than anticipated. As a result, all eligible patients were invited to participate (*N* = 220; 161 CHS and 59 NH). In total, 43 participants completed the survey, corresponding to an overall response rate of 20% (22% among CHS and 14% among NH participants). All eligible patients were also offered an interview. Of these, 8 participants completed interviews (5 CHS; 3 NH). Interviews were conducted across both health services until data saturation was reached and no new themes emerged [20].

### 2.5 Data Collection

Survey data were collected and managed using REDCap (Research Electronic Data Capture) electronic data capture tools hosted at CHS [21]. The online survey consisted predominantly of closed (multiple choice) questions. The survey included questions on demographics and health status, foot pain and HV severity measures, HV treatment, health‐related quality of life and depression, anxiety, and stress scales. The survey was initially piloted by CHS Community Care podiatry team to critique online usability and content. The final survey was adapted based on feedback, while upholding the requirements of standardised and licenced data collection tools including: Functional Comorbidity Index (FCI) [22], Manchester‐Oxford Foot Questionnaire (MOxFQ) [23], EuroQol 5‐dimension, 5‐level questionnaire (EQ‐5D‐5L) [24, 25], and Depression, Anxiety and Stress Scale −21 (DASS‐21) [26].

Patients who registered their interest in participating in an interview were contacted by study personnel and provided with a participant information sheet and consent form. Of the 13 CHS participants expressing interest, six proceeded to interview (three face‐to‐face, three via telephone), with one later excluded due to ineligibility (referral not attributed to HV). Of the 12 NH participants contacted, three completed interviews (one face‐to‐face, two online). Non‐participation was primarily due to loss of interest or time constraints. CHS patient interviews were conducted by KV (female), while NH interviews were conducted by MC (male). Interviews were semi‐structured with open ended questions. A question guide was developed in consultation with the research team and with consideration of a study that explored the lived experience of people with painful HV [9].

No relationship was established with the participants prior to the data collection process. At the start of interviews, interviewers provided background and discussed the purpose of the interview with participants. Recruitment continued until data saturation was reached across the dataset with no substantially new issues being identified relevant to the study aims and the participants' accounts reinforced issues identified in earlier interviews. Although fewer participants were recruited from Northern Health, the interviews from this site reflected the same issues identified across CHS, and no new concepts were emerging that suggested a need for further recruitment [17, 27]. All interviews lasted between 09:57 to 40:17 (mm:ss) with an average interview time of 27 minutes. No repeat interviews were conducted. Interviews were recorded and transcribed for data analysis.

### 2.6 Data Analysis

Quantitative statistical analysis was conducted using IBM SPSS Statistics version 29.0 (IBM Corp, NY, USA). Nominal data (e.g., ethnicity, education, and major medical conditions) were reported as frequencies (percentages). All continuous data were explored for normality using a combination of graphical outputs (histograms, box plots, P‐P plots, and Q‐Q plots) and statistical tests (Shapiro–Wilk, Kolmogorov–Smirnov, skewness, and kurtosis) and if normally distributed, reported as mean (standard deviation). No inferential comparisons were performed between sites due to the sample size.

Qualitative data were analysed using applied inductive thematic analysis [28], an approach focused on identifying patterns of meaning directly from the data in relation to the study aims. Interview recordings were transcribed verbatim by a research assistant. Familiarisation with the data occurred through repeated reading of the transcripts. Two researchers (KV and MC) independently conducted line by line coding using an inductive approach by applying codes to segments of text that captured ideas relevant to participants' experiences. The researchers then met to discuss and refine the coding framework before applying it to the remaining transcripts, focusing on the meaning and context of participants' responses. Similar codes were grouped into broader categories, which were reviewed and refined as the analysis progressed across the transcripts. This process allowed categories to be adjusted and new codes to be added as further insights emerged. Coding was conducted manually without the use of qualitative analysis software. Through ongoing discussion between KV and MC, categories were examined for patterns and developed into broader themes that reflected key aspects of the participants' experiences.

The qualitative analysis was undertaken by KV and MC, registered podiatrists with experience in clinical education and orthopaedic outpatient care. MC also works in an advanced practice podiatry role, involving the triage, assessment, and management of patients referred by general practitioners for specialist opinion. MC is also an experienced qualitative researcher. Their clinical backgrounds informed interpretation of the participants' stories, particularly in relation to treatment pathways and clinical decision‐making. Reflexive discussion was used throughout the analysis to consider how professional perspectives may have influenced theme development. A summary of themes was shared with interview participants for comment.

Using a convergent mixed‐methods design [17], the quantitative and qualitative data were analysed separately by the research team before being brought together for comparison. After completing the individual analyses, the results were examined side by side to identify areas where the two forms of data converged, complemented each other, or diverged highlighting aspects of the participants' experiences that were not evident in the other dataset. The aim was to merge the findings in a systematic way, allowing pattern comparison across the datasets.

## 3 Results

### 3.1 Survey

#### 3.1.1 Participant Characteristics

Participant characteristics are presented in Table 1. A total of 220 patients were invited to participate in the study, of whom 43 (20%) completed the survey (CHS 35; 22%); (NH 8; 14%). The median age of participants was 58 years, and the majority were female (84%). Forty percent (40%) of participants reported holding a pension or health care card. Participants from CHS reported a higher prevalence of self‐reported medical comorbidity compared with those from NH.

**TABLE 1:** Participant characteristics.

	Canberra (*n* = 35)	Northern (*n* = 8)	Total (*n* = 43)
Age, years (SD)	60.1 (11.5)	49.8 (15.4)	58.2 (12.8)
Sex at birth, male/female/non‐binary	6 (17.1)/28 (80.0)/1 (2.9)	1 (12.5)/7 (87.5)	7 (16.3)/35 (81.4)/1 (2.3)
Gender, male/female	6 (17.1)/29 (82.9)	1 (12.5)/7 (87.5)	7 (16.3)/36 (83.7)
Height, m (SD)	1.7 (0.1)	1.6 (0.1)	1.7 (0.1)
Weight, kg (SD)	76.4 (14.5)	73.5 (20.2)	75.9 (15.5)
Body mass index, kg/m^2^ (SD)	26.9 (4.5)	28.9 (7.0)	27.2 (4.9)
Country of birth			
Australia	23 (65.7)	7 (87.5)	30 (69.8)
New Zealand	3 (8.6)	—	3 (7.0)
United Kingdom and Ireland	2 (5.7)	—	2 (4.7)
Other	7 (20.0)	1 (12.5)	8 (18.6)
Ethnicity^a^			
Oceanian	16 (45.7)	4 (50.0)	20 (46.5)
North‐West European	14 (40.0)	—	14 (32.6)
Southern and Eastern European	3 (8.6)	2 (25.0)	5 (11.6)
Southern and Central Asian	2 (5.7)	0 (0.0)	2 (4.7)
North African and Middle Eastern	—	1 (12.5)	1 (2.3)
South‐east Asian	—	1 (12.5)	1 (2.3)
Highest level of education			
High school	7 (20.0)	4 (50.0)	11 (25.6)
Apprenticeship	1 (2.9)	—	1 (2.3)
Certificate	3 (8.6)	3 (37.5)	6 (14.0)
Diploma	5 (14.3)	—	5 (11.6)
Undergraduate degree	9 (25.7)	—	9 (20.9)
Postgraduate degree	10 (28.6)	1 (12.5)	11 (25.6)
Concession card			
Pensioner	10 (28.6)	2 (25.0)	12 (27.9)
Healthcare card	3 (8.6)	2 (25.0)	5 (11.6)
Seniors' healthcare card	1 (2.9)	—	1 (2.3)
Other	3 (8.6)	—	3 (7.0)
Unsure	1 (2.9)	1 (12.5)	2 (4.7)
None	17 (48.6)	3 (37.5)	20 (46.5)
Self‐reported medical conditions			
Arthritis	17 (48.6)	2 (25.0)	19 (44.2)
Osteoporosis	4 (11.4)	2 (25.0)	6 (14.0)
Degenerative disc disease	9 (25.7)	—	9 (20.9)
Pulmonary disease	7 (20.0)	—	7 (16.3)
Myocardial infarction	2 (5.7)	—	2 (4.7)
Heart failure	1 (2.9)	—	1 (2.3)
Neurological disease	2 (5.7)	—	2 (4.7)
Cerebrovascular accident	1 (2.9)	—	1 (2.3)
Peripheral vascular disease	1 (2.9)	—	1 (2.3)
Gastrointestinal disease	1 (2.9)	—	1 (2.3)
Depression	8 (22.9)	2 (25.0)	10 (23.3)
Anxiety	6 (17.1)	1 (12.5)	7 (16.3)
Visual impairment	5 (14.3)	1 (12.5)	6 (14.0)
Hearing impairment	3 (8.6)	—	3 (7.0)
Smoking status—previous daily	7 (20.0)	2 (25.0)	9 (20.9)
Smoking status—current daily	3 (8.6)	—	3 (7.0)

*Note: n* (%) unless otherwise specified.

Abbreviation: SD = standard deviation.

^a^ Ethnicity documented using the Australian Standard Classification of Cultural and Ethnic Groups.

#### 3.1.2 Hallux Valgus Characteristics

Survey respondents reported long‐standing HV, with 47% experiencing associated pain for more than 6 years. Self‐reported severity using the Manchester scale was most rated as moderate or severe, with 42% reporting bilateral symptoms. Mean scores on the MOxFQ indicated moderate impairment across pain, walking/standing, and social interaction domains (Table 2).

**TABLE 2:** Hallux valgus characteristics.

	Canberra (*n* = 35)	Northern (*n* = 8)	Total (*n* = 43)
Pain duration			
< 1 year	1 (2.9)	—	1 (2.9)
1–2 years	—	—	—
3–4 years	5 (14.3)	3 (37.5)	8 (18.6)
5–6 years	4 (11.4)	2 (25.0)	6 (14.0)
> 6 years	20 (57.1)	—	20 (46.5)
No pain	4 (11.4)	1 (12.5)	5 (11.6)
Unsure	1 (2.9)	—	1 (2.9)
Manchester scale			
Right foot			
None	5 (14.3)	—	5 (11.6)
Mild	5 (14.3)	1 (12.5)	6 (14.0)
Moderate	15 (42.9)	4 (50.0)	19 (44.2)
Severe	10 (28.6)	3 (37.5)	13 (30.2)
Left foot			
None	6 (17.1)	2 (28.6)	8 (19.0)
Mild	4 (11.4)	1 (14.3)	5 (11.9)
Moderate	11 (31.4)	3 (42.9)	14 (33.3)
Severe	14 (40.0)	1 (14.3)	15 (35.7)
Bilateral hallux valgus symptoms	17 (48.6)	1 (12.5)	18 (41.9)
MOxFQ—right foot, mean (SD)			
Summary index score	54.2 (18.5)	54.7 (18.1)	54.3 (18.2)
Walking/standing	56.6 (21.1)	56.5 (32.5)	56.6 (23.0)
Pain	52.5 (18.0)	49.2 (13.9)	51.9 (17.2)
Social interaction	52.2 (24.0)	58.3 (11.6)	53.3 (22.2)
MOxFQ—left foot, mean (SD)			
Summary index score	48.9 (22.5)	72.9 (15.8)	51.6 (23.0)
Walking/standing	47.3 (25.3)	84.5 (20.9)	51.5 (27.3)
Pain	48.3 (21.6)	60.0 (5.0)	49.6 (21.0)
Social interaction	52.3 (28.4)	68.8 (27.2)	54.2 (28.2)

*Note: n* (%) unless otherwise specified.

Abbreviations: MOxFQ = Manchester‐Oxford Foot Questionnaire, SD = standard deviation.

#### 3.1.3 Hallux Valgus Consultation Characteristics

Pain (91%) and shoe‐fitting difficulties (81%) were the most reported reasons for seeking care. General practitioners were the primary health professional consulted (88%), while 35% of CHS participants reported consulting a podiatrist. Nearly 70% of participants believed surgery was necessary, although fewer expected to undergo surgery (44%). Most participants reported that their HV had worsened while waiting for orthopaedic consultation (65%). Consultation characteristics are summarised in Table 3.

**TABLE 3:** Hallux valgus consultation characteristics—*n* (%).

	Canberra (*n* = 35)	Northern (*n* = 8)	Total (*n* = 43)
Reasons for seeking treatment			
Pain	32 (91.4)	7 (87.5)	39 (90.7)
Appearance	17 (48.6)	5 (62.5)	22 (51.2)
Shoe‐fitting problems	27 (77.1)	8 (100.0)	35 (81.4)
Activity limitations	22 (62.9)	4 (50.0)	26 (60.5)
Health professional consultation	35 (100.0)	7 (87.5)	42 (97.7)
General practitioner	34 (97.1)	4 (50.0)	38 (88.4)
Podiatrist	15 (42.9)	—	15 (34.9)
Pharmacist	—	2 (25.0)	2 (4.7)
Physiotherapist	1 (2.9)	1 (12.5)	2 (4.7)
Other	3 (8.6)	—	3 (7.0)
Management			
Imaging	7 (20.0)	3 (37.5)	10 (23.3)
Education	8 (22.9)	4 (50.0)	12 (27.9)
Surgical beliefs			
Need surgery	22 (62.9)	8 (100.0)	30 (69.8)
Expect to have surgery	14 (40.0)	5 (62.5)	19 (44.2)
How long have you been waiting for an orthopaedic consultation?			
< 1 year	5 (14.3)	—	5 (11.6)
1–2 years	5 (24.3)	6 (75.0)	11 (25.6)
3–4 years	9 (25.7)	1 (12.5)	10 (23.3)
5–6 years	9 (25.7)	1 (12.5)	10 (23.3)
7–8 years	1 (2.9)	—	1 (2.3)
> 8 years	6 (17.1)	—	6 (14.0)
While waiting to see an orthopaedic surgeon for your bunion, has the condition of your bunion changed?			
Improved	1 (2.9)	—	1 (2.3)
Unchanged	10 (28.6)	1 (12.5)	11 (25.6)
Worsened	21 (60.0)	7 (87.5)	28 (65.1)
Unsure	3 (8.6)	—	3 (7.0)
While waiting to see an orthopaedic surgeon for your bunion, would accept an appointment with a podiatrist	28 (80.0)	5 (62.5)	33 (76.7)

#### 3.1.4 Health‐Related Quality of Life and Psychological Health

All participants reported some level of mobility limitation on the EQ‐5D‐5L. The majority reported slight to moderate pain or discomfort, and approximately half reported some degree of anxiety or depression. Mean scores on DASS‐21 for depression, anxiety, and stress fell within normal ranges at the group level. Self‐rated overall health on the EQ visual analogue scale was moderate, with substantial variability between individuals (Table 4).

**TABLE 4:** Health‐related quality of life, depression, anxiety and stress.

	Canberra (*n* = 35)	Northern (*n* = 8)	Total (*n* = 43)
EQ‐5D‐5L			
Mobility			
No problems	—	—	—
Slight problems	9 (25.7)	—	9 (20.9)
Moderate problems	15 (42.9)	4 (50.0)	19 (44.2)
Severe problems	7 (20.0)	3 (37.5)	10 (23.3)
Extreme problems/unable to do	4 (11.4)	1 (12.5)	5 (11.6)
Self‐care			
No problems	29 (82.9)	7 (87.5)	36 (83.7)
Slight problems	4 (11.4)	—	4 (9.3)
Moderate problems	2 (5.7)	1 (12.5)	3 (7.0)
Severe problems	—	—	—
Extreme problems/unable to do	—	—	—
Usual activities			
No problems	16 (45.7)	4 (50.0)	20 (46.5)
Slight problems	9 (25.7)	1 (12.5)	10 (23.3)
Moderate problems	7 (20.0)	2 (25.0)	9 (20.9)
Severe problems	2 (5.7)	1 (12.5)	3 (7.0)
Extreme problems/unable to do	1 (2.9)	—	1 (2.3)
Pain/discomfort			
No pain or discomfort	2 (5.7)	—	2 (4.7)
Slight pain or discomfort	13 (37.1)	3 (37.5)	16 (37.2)
Moderate pain or discomfort	15 (42.9)	3 (37.5)	18 (41.9)
Severe pain or discomfort	4 (11.4)	2 (25.0)	6 (14.0)
Extreme pain or discomfort	1 (2.9)	—	1 (2.3)
Anxiety/depression			
I am not anxious or depressed	16 (45.7)	4 (50.0)	20 (46.5)
I am slightly anxious or depressed	7 (20.0)	1 (12.5)	8 (18.6)
I am moderately anxious or depressed	10 (28.6)	2 (25.0)	12 (27.9)
I am severely anxious or depressed	2 (5.7)	1 (12.5)	3 (7.0)
I am extremely anxious or depressed	—	—	—
Visual analogue scale, mean (SD)	67.0 (22.1)	64.6 (22.3)	66.6 (21.9)
DASS‐21, mean (SD)			
Depression	4.5 (4.3)	5.9 (5.8)	4.8 (4.6)
Anxiety	3.5 (3.4)	3.0 (3.0)	3.4 (3.3)
Stress	5.3 (4.8)	5.3 (4.3)	5.3 (4.7)

*Note: n* (%) unless otherwise specified.

Abbreviations: DASS‐21 = depression, anxiety and stress scale, SD = standard deviation.

### 3.2 Interviews

#### 3.2.1 Participant Characteristics

Eight participants took part in semi‐structured interviews (5 CHS; 3 NH). Most participants were female (75%), with the cohort overall having a median age of 67 years (range 35–80 years). Interview participants typically reported longer symptom duration and greater cumulative impact than survey respondents. Most participants had been living with HV for more than a decade and had experienced prolonged periods awaiting specialist review.

#### 3.2.2 Hallux Valgus Characteristics

Interview participants typically described HV duration ranging from approximately 10 to 20 years, with self‐reported severity most rated as moderate to severe. Onset was mostly gradual, with many participants reporting delayed help‐seeking until pain or functional limitation became difficult to manage. Perceived contributing factors included footwear, family history, and age‐related changes, although several participants expressed uncertainty about the underlying cause of their condition. Bilateral involvement and progressive symptom worsening over time were frequently reported.

#### 3.2.3 Qualitative Themes

An inductive applied thematic analysis identified four interpretive themes that reflected participants' experiences of living with HV and navigating care while awaiting orthopaedic consultation: (1) The biopsychosocial burden of living with HV; (2) Self‐managing in the absence of guidance; (3) Navigating fragmented and uncertain care pathways; and (4) Seeking understanding, validation, and informed decision‐making. These themes illustrate an interconnected journey whereby participants experienced substantial burden, relied on self‐directed management, encountered challenges accessing care, and sought greater understanding and support from health services.

Qualitative data were organised in tabular form to enable comparison across participants (Supporting Information S1). Participants were provided with a summary of the identified themes for review. Feedback from three participants (*N* = 2 from CHS and *N* = 1 from NH) did not result in any substantive changes to the thematic structure.

##### 3.2.3.1 Theme 1: The Biopsychosocial Burden of Living With HV

The impact of HV was described as profound and pervasive, shaping participants' physical function, emotional wellbeing, and everyday decision‐making. Pain was almost universal, characterised as constant, intrusive, and often severe. Pain occurred both during weight‐bearing and at rest, with sleep disruption commonly reported. As one participant explained, “…I get the shooting pains even when I am lying in bed at night.” [#7], highlighting the persistent nature of symptoms.

Footwear restriction emerged as a dominant and distressing consequence, marked by ongoing tension between comfort, stability, and social acceptability. For some, footwear changes had broader functional consequences. One participant linked orthotics and shoe modifications to a fall and subsequent loss of confidence, stating, “Because of the bunions I had to get shoes, orthotics, and then I fell over… Since then, I lost a lot of confidence walking” [#6]. Others described embarrassment and social constraint, “Occasionally you do need to go to a wedding or something and you can't wear flat shoes” [#2], highlighting the influence on social participation.

Most participants restricted walking and weight‐bearing activities, often reluctantly, leading to cumulative physical and psychosocial effects. Reduced activity was associated with weight gain and altered movement patterns, as illustrated by one participant: “I can't walk for long distances. I put on about 10 kilos” [#2], and another who stated, “I'm changing the way I walk to suit the pain in my foot” [#1]. Beyond physical consequences, the long‐term burden of “chronic pain” [#4] was described as mentally exhausting and emotionally disruptive. Participants reported frustration, low mood, depression, self‐consciousness, and negative body image as one participant described:

It affects your life because you just, I suppose we are all a bit vain you know, so you just look at it [HV] and you hate it. You hate yourself. So, it affects you psychologically too

[#2]

Collectively, participants described HV as extending far beyond a foot deformity, affecting multiple aspects of their lives and creating an ongoing physical, psychological, and social burden driving a desire to find coping strategies and treatment options.

##### 3.2.3.2 Theme 2: Self‐Managing in the Absence of Guidance

Coping with HV was largely described as self‐directed and evolved through trial and error in the context of perceived limited professional guidance. Participants described experimenting with strategies over time rather than following structured conservative management plans. Footwear modification was the most common response, driven by necessity rather than informed recommendation. Participants reported limited and inconsistent footwear preferences with choices including athletic footwear, boots, thongs, slippers, and one participant describing medical grade footwear. As one participant explained, coping meant “…wearing only horrible flat shoes, one size or two sizes bigger because sometimes they are not wide enough” [#2]. These pragmatic adaptations highlight how symptom management was explored independently, with comfort primarily prioritised over aesthetics.

Activity modification commonly accompanied footwear changes. Participants described reducing walking distances, avoiding prolonged standing, or withdrawing from valued activities to prevent pain flares. While framed as protective, these strategies were expressed by most as costly, with one participant stating, “It's easier to modify my lifestyle to be less active than I was, but I dislike that” [#4]. Pain was described as a signal to stop activity, reflecting a cautious approach shaped by fear of deterioration rather than clinician input. However, pharmacological strategies were used sparingly and with caution, as participants weighed relief against perceived risks including side effects, dependency, and symptom masking. As one participant explained:

When it gets excruciating, I just have to stop and sit down. Whereas I think if I'm taking painkillers, I'll work, I'll walk through it and make it worse

[#1]

Exposure to structured education or conservative management was limited across participants, resulting in a reliance on self‐management and self‐education. Participants frequently sought information online to understand their condition and explore options, although most expressed scepticism of online products. As one participant explained, “I search through the internet for this thing [splint]… I don't know how good they are” [#8]. With a perceived lack of options, many participants described a process of adapting to persistent symptoms, often marked by resignation but also resilience.

I would say it's the bane of my life, but because it's a form of chronic pain eventually you get to the point where I think you sort of despair of doing anything about it. So, you just kind of work around it

[#4]

Overall, many participants described adapting to persistent symptoms through a combination of resignation and resilience, accepting ongoing limitations while continuing to search for ways to maintain function and independence.

##### 3.2.3.3 Theme 3: Navigating Fragmented and Uncertain Care Pathways

Participants consistently described their interactions with health services as fragmented, procedural, and minimally informative, particularly at first presentation and during extended periods on orthopaedic waitlists. General practitioners were the universal first point of contact, however consultations were often reported as brief and referral‐focused, with limited physical examination, imaging, or education. As one participant reflected, “they [GP] just said OK, probably you need to see a specialist. Didn't look at my feet or anything.” [#2].

Beyond referral, participants described uncertainty and poor communication regarding specialist pathways, particularly within the public system. Understanding of referral status, prioritisation, and anticipated timeframes appeared limited for both patient and GPs, with one participant reporting, “To be honest, when you told me I was still on the waitlist, I was really surprised. I thought maybe that had somehow expired” [#4]. The responsibility for navigating the process was frequently perceived to rest with the patient alone. This was clearly illustrated by the recollection of one participant:

They [GP] said, ‘just keep waiting’, and then I went and called up the waitlist, and they said, ‘you should talk to your GP to get another referral’. So, I went back to the GP, and they said, ‘I don’t know why they are asking this, but here you go’…

[#7]

While awaiting specialist care, participants commonly described worsening pain, deformity, and functional decline. Experiences with allied health, particularly podiatry, were mixed. Some reported benefit from podiatric management, “He [the podiatrist] made them [toe spacers] specially and it's good because if I don't wear them, I can feel the difference” [#5]. While others perceived encounters as transactional or poorly explained, “They [podiatrist] tried to sell me some inserts for my shoes” [#1]. Financial constraints strongly shaped engagement with care. One participant explained, “Well, I'm on a concession card and I just don't have the disposable income” [#6], highlighting the reliance on public waitlists despite symptom progression.

Together, these experiences reflected a broad sense of uncertainty and fragmentation within the care pathway, leaving patients feeling responsible for navigating complex healthcare processes while managing worsening symptoms.

##### 3.2.3.4 Theme 4: Seeking Understanding, Validation, and Informed Decision‐Making

Participants described reasonable expectations of orthopaedic consultation, centred on gaining clarity rather than pursuing immediate surgical intervention. While surgery was acknowledged as a possible outcome, it was rarely viewed as the primary goal. Instead, participants sought authoritative assessment and explanation to contextualise their condition and inform decision making. As one participant explained:

It’s not like I’m waiting for surgery, because I may or may not think that’s a good idea depending on what the person [specialist] says

[#4]

This reflected an expectation that orthopaedic review would fill gaps left by earlier care, particularly through clear explanation and informed discussion of management options.

A consistent expectation across participants was the importance of thorough clinical examination as the foundation of care. Hands‐on assessment was viewed as essential for diagnosis and as a sign that concerns were being taken seriously, as demonstrated by one participant's comment: “A proper physical examination would make me feel like they're taking it seriously” [#1]. Beyond diagnosis, participants wanted education about prognosis, symptom progression, and the anticipated outcomes of both conservative and surgical options. This included realistic, individualised information to support shared decision making, as illustrated by the request, “What would be better for me? Is it [surgery] going to help me 100%? … tell me what the effects are going to be” [#9].

Feeling heard and validated was closely linked to both examination and education, highlighting the importance of clear, two‐way communication. Participants valued honesty and realism over reassurance, with one stating:

I just want someone to be straight with me about what’s actually going on…If they can’t fix it, just tell me that. I’d rather know than be given false hope

[#1]

All participants were receptive to podiatry‐led models of care while awaiting orthopaedic review, particularly for assessment, education, and guidance on conservative and self‐management strategies. However, this was contingent on care being evidence‐based and explanatory rather than transactional. As one participant explained, “I'd see a podiatrist if it was about assessment and advice, not just selling something” [#2].

Overall, participants sought care that fostered understanding, trust, and informed decision‐making, highlighting a desire for collaborative and person‐centred care rather than a sole focus on surgical intervention.

### 3.3 Integrated Findings

#### 3.3.1 Impact of Hallux Valgus

Integration of quantitative and qualitative findings demonstrated strong convergence regarding the substantial impact of HV. Survey data showed moderate impairment in pain and walking/standing, along with widespread mobility limitations and reduced quality of life. Interview findings provided depth and context to these results, illustrating how persistent pain, footwear restriction, activity avoidance, and sleep disturbance permeated daily life and contributed to emotional distress. While average psychological symptom scores were low at the group level, qualitative accounts highlighted considerable individual variability and psychosocial burden not fully captured by aggregate measures.

#### 3.3.2 Coping Strategies and Unmet Needs

Quantitative findings indicated low levels of formal education and conservative management, while qualitative data revealed reliance on self‐directed coping strategies within a context of inconsistent guidance and uncertainty. Together, the findings suggest a mismatch between patient needs and access to structured, evidence‐based non‐surgical support, contributing to frustration, resignation, and prolonged symptom burden.

#### 3.3.3 Health Service Experience and System Navigation

Both datasets highlighted challenges in health‐service navigation. Survey data confirmed that general practitioners were the primary entry point to care and that long waiting times for specialist consultation were common. Qualitative findings expanded on this by revealing limited primary assessment, poor communication, and lack of transparency regarding waitlists. The convergence of findings indicates that prolonged waiting contributes directly to worsening symptoms, reduced function, and regret over missed opportunities for earlier intervention.

#### 3.3.4 Expectations and Acceptability of Alternative Care Models

Although many survey participants believed surgery was necessary, fewer expected to proceed with surgery, reflecting ambivalence rather than clear surgical intent. Interview data clarified this discrepancy, showing that patient priorities centred on assessment, explanation, and personalised advice rather than surgery alone. Strong convergence was evident regarding acceptability of podiatry‐led care, with most participants willing to engage with podiatry services while awaiting orthopaedic review, provided care was perceived as trustworthy and individualised.

Overall, the integrated findings suggest that people with HV experience substantial and prolonged burden while awaiting orthopaedic consultation, alongside unmet needs for education, reassurance, and conservative management. Summarised data integration is revealed in table format in Table S1.

## 4 Discussion

This mixed‐methods study explored the experiences, needs, perceptions, and expectations of people with HV awaiting orthopaedic consultation within two Australian public health services. By integrating data with in‐depth qualitative interviews, this study provides a nuanced understanding of the HV patient journey during prolonged surgical wait times and identifies key opportunities for service redesign. Overall, the findings highlight prolonged symptom duration before referral, substantial physical and psychosocial burden while waiting, limited access to and understanding of non‐surgical management, and strong patient receptivity to podiatry‐led models of care.

A key finding was the long duration of HV symptoms, with nearly half of survey respondents reporting pain duration greater than 6 years and interview participants describing symptoms spanning one to two decades. This aligns with earlier epidemiological work demonstrating the progressive nature of HV and its cumulative impact on function and quality of life [1, 2]. Delayed help‐seeking may reflect the perception of HV as a benign or cosmetic issue until pain becomes unmanageable, a belief reinforced by inconsistent messaging from primary care providers and limited access to early intervention [6]. These findings suggest that opportunities for assessment and conservative intervention may not always occur early in the disease trajectory. Earlier engagement with evidence‐based care could provide an opportunity to address symptoms and functional limitations before they become entrenched, although the effectiveness of such approaches in this population warrants further investigation.

Interview participants frequently linked altered gait patterns and activity avoidance to broader musculoskeletal discomfort and reduced physical function, consistent with previous evidence [4, 29]. These accounts suggest that the consequences of HV may extend beyond localised foot symptoms and influence mobility, participation in physical activity, and overall wellbeing. While the extent of these relationships requires further investigation, the findings highlight the potential value of timely assessment and access to appropriate management strategies to address the wider impacts of the condition.

Consistent with the study aim, integrated findings demonstrate that HV has a multidimensional impact on patients awaiting orthopaedic review. Participants reported moderate impairment across MOxFQ pain, walking/standing, and social interaction domains, while EQ‐5D‐5L results indicated mobility limitations. These findings are consistent with previous studies demonstrating the negative impact of HV on quality of life and functional ability [2, 3, 4, 5]. Qualitative data provided additional insight into how these impairments were experienced in everyday life, with participants describing chronic pain, sleep disturbance, footwear restriction, activity avoidance, social limitations, and negative self‐perceptions. Importantly, the cumulative burden extended beyond symptoms alone, affecting confidence, participation in valued activities, and emotional wellbeing. These findings suggest that the impact of HV is not solely biomechanical but has broader implications for social participation, self‐identity, and overall wellbeing. Consequently, approaches to HV management that focus exclusively on structural deformity may overlook aspects of the condition that patients perceive as most important.

Although mean DASS‐21 scores fell within normal ranges, approximately half of participants reported some level of anxiety or depression on the EQ‐5D‐5L, and qualitative interviews revealed considerable emotional burden among some individuals. Rather than indicating an absence of psychological impact, these findings highlight the heterogeneity of patient experiences and the value of qualitative inquiry in identifying aspects of living with HV that may not be fully captured by group‐level summary measures. Interview participants described frustration, hopelessness, reduced confidence, negative body image, and emotional fatigue associated with persistent symptoms and prolonged waiting. However, it is also possible that individuals experiencing a greater burden from their condition were more likely to volunteer for interviews, which may have increased representation of these experiences within the qualitative findings. Nevertheless, the data suggest that the psychological impact of HV may be clinically important for at least a subgroup of patients and warrants greater consideration during assessment and management. This reinforces the importance of person‐centred care that acknowledges both the physical and emotional consequences of the condition.

These findings mirror Cotchett et al. [9], who described HV as a condition that permeates daily decision‐making, self‐perception, and psychosocial wellbeing. Taken together, the present findings and previous qualitative work indicate that patients may benefit from greater validation, supportive communication, and opportunities to discuss the broader consequences of their condition, particularly during prolonged periods awaiting specialist review [30]. Participants described coping with HV largely through self‐directed and trial‐and‐error approaches, mostly footwear changes and activity modification. While these strategies sometimes provided symptom relief, they were frequently accompanied by frustration, inconsistent advice, and uncertainty. This finding is consistent with previous reports of variability in conservative HV management and limited implementation of available evidence‐based interventions [6, 8]. Relatively few participants reported receiving structured education, exercise prescription, or orthotic management despite emerging evidence supporting such interventions for pain reduction and functional improvement [7, 8].

The reliance on self‐management may reflect gaps in both patient education and access to conservative care within existing management pathways. Participants often sought information independently and described uncertainty regarding which interventions were worthwhile, highlighting a need for clearer guidance and shared decision‐making. Distrust of commercially driven care further emphasised the importance of transparent communication regarding the expected benefits and limitations of conservative management strategies. Collectively, these findings suggest that patients are seeking not only treatment, but also credible information and support to make informed decisions about their care.

Difficulties navigating the health system were a central theme. GPs were the primary first point of contact, consistent with national data [6], yet participants frequently reported limited assessment, imaging, and education. Referral to orthopaedics was often perceived as occurring with little discussion of alternative management options, and many participants reported uncertainty regarding referral status, prioritisation processes, and anticipated waiting times. These findings align with previous research demonstrating limited use of non‐surgical interventions prior to orthopaedic referral [10].

Prolonged wait times, particularly within CHS, were associated with perceived symptom progression and functional decline. However, shorter wait times at NH did not translate into improved patient or GP understanding of referral pathways or service navigation. This suggests that patient experiences may be influenced not only by waiting time itself but also by the quality of communication and support provided while waiting. Improving transparency around referral pathways, expected timeframes, and available management options may therefore represent an important opportunity to improve patient experience irrespective of waitlist duration.

Despite extended waits, participants' expectations of orthopaedic consultation were generally modest and centred on assessment, diagnosis, prognosis, and education rather than surgery. While a majority believed surgery was necessary, fewer expected to proceed, reflecting ambivalence and uncertainty consistent with prior qualitative work [31]. Importantly, participants expressed a strong desire to feel listened to, validated, and provided with personalised information, needs that could be met earlier in the care pathway.

A key finding was that over three‐quarters of participants indicated they would accept an appointment with a podiatrist while awaiting orthopaedic review. Previous studies have demonstrated that advance practice podiatry‐led models can reduce orthopaedic demand while maintaining high levels of patient satisfaction [12, 14, 15, 16]. However, the present study did not evaluate service effectiveness. Rather, the findings demonstrate strong patient acceptability of podiatry‐led care and identify several unmet needs, including assessment, education, communication, and conservative management, that may be amenable to such approaches. Consequently, advanced practice podiatry warrants further evaluation as a strategy to address these needs within HV care pathways. Qualitative findings further suggest that patient trust may depend on perceptions of clinical independence, expertise, and provision of evidence‐based advice rather than commercial product sales.

Overall, the findings suggest that advanced practice podiatry models may offer a promising approach to addressing unmet needs within HV care pathways, particularly for triage, early assessment, education, and non‐surgical management, aligning with broader musculoskeletal service reform strategies [32, 33]. Additionally, the findings also highlight the potential value of improving communication and transparency across referral pathways. Greater information sharing between specialist services, primary care providers, and patients may reduce uncertainty, support informed clinical decision‐making, and improve patient experience while awaiting specialist review. Future research is needed to determine whether advanced practice podiatry models can effectively address these unmet needs while improving patient outcomes and service delivery.

Strengths of this study include its mixed‐methods design, use of validated measures, and integration of patient voices to contextualise quantitative findings. The inclusion of participants from two public health services enabled exploration of common experiences across different service contexts.

Several limitations should be acknowledged. First, the survey response rate was relatively low, increasing the possibility of response bias. Second, interview participants may also have differed from non‐participants in important ways, including their motivation to discuss their experiences or the degree to which symptoms affected them. In keeping with qualitative research principles, the aim of this study was not statistical generalisation. Instead, sufficient contextual detail has been provided to allow readers to consider the potential transferability of findings to other healthcare settings. Finally, as participants were recruited from Australian public health services with prolonged orthopaedic waiting times, experiences may differ in alternative healthcare systems or settings.

## 5 Conclusion

This study revealed that HV imposes a substantial and prolonged burden on patients awaiting orthopaedic consultation, with unmet needs persisting across physical, psychosocial, and informational domains. Patients are not universally seeking surgery but are actively seeking understanding, validation, and management options. Podiatry‐led triage and advanced practice models represent a promising, patient‐centred approach to improving care pathways for HV and addressing the challenges of growing orthopaedic waitlists.

## Author Contributions

**Katja Veenendaal:** data curation, formal analysis, writing – original draft. **Matthew Cotchett:** data curation, formal analysis, writing – review and editing. **Hylton B. Menz:** formal analysis, writing – review and editing.

## Funding

This research was funded by Canberra Health Services.

## Ethics Statement

This study was approved by the ACT Health Human Research and Ethics Committee (2024.LRE.00317). Ethics approval was also obtained via cross institutional approval with Northern Health (SSA/116605/NH‐2025) and University of Canberra (UC) (14440).

## Consent

All participants provided informed consent prior to data collection.

## Conflicts of Interest

The authors declare no conflicts of interest.

## Permission to Reproduce Material From Other Sources

The authors have nothing to report.

## Supporting information


Supporting Information S1

**Table S1:** Integrated qualitative themes and quantitative findings.

## Data Availability

Data are available from the authors on reasonable request.
