# Supplementary material for: Perceptions and Experience of Patients Awaiting Orthopaedic Consultation for Hallux Valgus in Two Public Health Services in Australia
Source: J Foot Ankle Res. 2026 Sep 12;19(3):e70209. doi: 10.1002/jfa2.70209 (PMC13570671; doi:10.1002/jfa2.70209)
Supplement: Supplementary file 1 [file JFA2-19-e70209-s001.docx]

**Supplementary file 2**

**Table A1.** Integrated qualitative themes and quantitative findings

| **Theme** | **Quantitative findings** | **Qualitative findings** | **Integrated summary** |
| --- | --- | --- | --- |
| **Impact of hallux valgus** | Moderate impairment on MOxFQ pain and walking/standing. Universal mobility limitations on EQ‑5D‑5L. Anxiety/depression reported by ~50%; mean DASS‑21 within normal range. | Persistent pain, footwear restriction, reduced activity, sleep disturbance, and psychosocial distress. | Strong agreement regarding physical and functional burden; psychosocial impact varied across individuals. |
| **Coping strategies** | Low reported exposure to education and conservative management. | Predominantly self‑directed strategies (footwear changes, activity reduction, cautious analgesia) with limited professional guidance. | Limited access to structured non‑surgical care resulted in reliance on self‑management. |
| **Health service experience** | GPs as primary entry point; long wait times common; most reported worsening while awaiting review. | Limited assessment and education in primary care, unclear waitlist processes, and symptom progression while waiting. | Health system processes contributed to delayed intervention and symptom progression. |
| **Expectations of care** | More believed surgery was needed than expected to proceed; >75% would accept podiatry while waiting. | Desire for assessment, explanation, and personalised advice rather than surgery alone; acceptance of podiatry‑led care. | Support for alternative care models focused on education and conservative management. |
